# Prevalence of cryptococcal infection among advanced HIV patients in Argentina using lateral flow immunoassay

**DOI:** 10.1371/journal.pone.0178721

**Published:** 2017-06-15

**Authors:** Claudia Frola, Liliana Guelfand, Gabriela Blugerman, Edgardo Szyld, Sara Kaufman, Pedro Cahn, Omar Sued, Héctor Pérez

**Affiliations:** 1 Service of Infectious Diseases, Juan A. Fernández Hospital, Buenos Aires, Argentina; 2 Clinical Research Department, Fundación Huésped, Buenos Aires, Argentina; 3 Microbiology Laboratory, Juan A. Fernández Hospital, Buenos Aires, Argentina; 4 Oklahoma University Health Sciences Center, Oklahoma City, Oklahoma, United States of America; RIVM, NETHERLANDS

## Abstract

**Background:**

Globally, Latin America ranks third among regions with most cases of AIDS related cryptococcal meningitis. In 2009, a lateral flow immunoassay (LFA) for the detection of cryptococcal antigen (CrAg) was developed as a potential point-of-care test for diagnosis of cryptococcal infection. In 2011 World Health Organizations recommended on CrAg screening for HIV positive persons with CD4 below 100 cells/μL, followed by preemptive fluconazole treatment. However, in Argentina no formal recommendations for CrAg screening have been issued.

**Methods:**

HIV positive patients > = 18 years with advanced immunosuppression (CD4 counts ≤100 cells/μL within 3 months or WHO stage III/IV), who visited the hospital between April 1, 2014 and January 31, 2015, were included. The LFA was performed according to the manufacturer’s instructions on all serum samples. When CrAg detection was positive, a lumbar puncture was performed to rule out cryptococcal meningitis. Patients without evidence of meningeal involvement were treated with preemptive oral fluconazole in ambulatory care.

**Results:**

We included 123 patients. Prevalence of CrAg-positivity was 8.1%. Among the 10 CrAg-positive patients, 6 had meningeal involvement detected through the CSF analysis (CSF India-ink testing, CSF CrAg and culture). The remaining 4 patients with positive CrAg received targeted preemptive treatment with oral fluconazole and were free of cryptococcal disease during the follow-up period. None of the 113 patients with a negative CrAg test result developed cryptococcal disease.

**Conclusions:**

This is the first study in Argentina, to our knowledge, describing the prevalence of cryptococcosis and usefulness of CrAg screening. LFA provided early diagnosis to determine a high prevalence of CrAg in our hospital, and that screening for subclinical infection with preemptive antifungal treatment, prevented a substantial proportion of meningeal disease.

## Introduction

Globally, Latin America ranks third among regions with the most cases of AIDS related cryptococcal meningitis (CM) [1].

The HIV epidemic is currently stable in Argentina, with an estimated 126,000 infected people of which, 30% are unaware of their serostatus. The most recent epidemiological report showed changes in the distribution of new cases with a trend toward an increase of delayed diagnosis and the risk for subsequent opportunistic infections [2].

High fungal burden and altered mental status are the most important drivers of acute cryptococcal-related mortality, events that often occur in patients with late diagnosis [3,4]. The diagnosis of CM is usually made by lumbar puncture and India-ink testing of cerebrospinal fluid (CSF). However, the presenting symptoms of headache and fever are nonspecific, and lumbar puncture is often deferred until the disease progressed, resulting in worsening prognosis [5]. In 2009, a lateral flow immunoassay (LFA) for the detection of cryptococcal antigen (CrAg) was developed by IMMY (Immuno-Mycologics, Inc., OK, USA) as a potential point-of-care test for diagnosis of cryptococcal infection. This test is stable at room temperature (20–25°C), has a rapid turnaround time, requires very little technical skills and can be performed with minimal laboratory infrastructure. Its sensitivity is almost 100% with both serum and CSF samples [6].

The use of preemptive therapy in asymptomatic cases with positive antigenemia is not well defined, but international recommendation suggest its use based on expert opinion [7]. In 2011 World Health Organizations (WHO) recommended on CrAg screening for HIV positive persons with CD4 below 100 cells/μL, followed by preemptive fluconazole treatment in settings with high prevalence [8]. However, in Argentina no formal recommendations for CrAg screening have been issued.

CM management require complex treatments and usually prolonged hospitalizations, representing a significant increase in health costs. Treatment of asymptomatic or latent cryptococcal infection with oral fluconazole is a much less expensive and highly available option compared to standard-of-care for meningitis [5,9].

Our hypothesis is that routine serological testing would allow early detection of asymptomatic infected subjects. Therefore, the aim of this study was to evaluate the effectiveness of routine CrAg screening in patients with advanced HIV infection and to assess the benefits of preemptive treatment for the positive cases.

## Materials and methods

### Ethics statement

This study was approved by the institutional ethics committee of Juan A. Fernandez Hospital.

### Participants and study design

This prospective cohort study was conducted at Juan A. Fernandez Hospital, one of the main referral centers for HIV care in Buenos Aires City, Argentina with 3,951 HIV positive patients in active follow-up. After obtaining written informed consent, blood samples were drawn from eligible patients who visited the hospital between April 1, 2014 and January 31, 2015.

Included subjects were HIV positive, at least 18 years of age and presenting with advanced immunosuppression (CD4 counts ≤100 cells/μL within 3 months or WHO stage III/IV). We excluded patients with undetectable viral load within the previous 3 months, those with a diagnosis of cryptococcal disease within the previous year, or those receiving antifungal treatment within the last 14 days.

The following variables were collected from medical records and/or patient interviews and recorded on a case report form and transcribed to an Excel^®^ spreadsheet: age, gender, time of HIV diagnosis at enrolment, CD4 cell count, highly active antiretroviral therapy (HAART) status, viral load, WHO clinical stage, previous opportunistic infection, current or previous medication and current neurologic symptoms (e.g., headache, suspected meningitis). Clinical information of those patients with cryptococcosis was also collected with regard to site of cryptococcal infection (i.e. meningeal vs extra-meningeal), type of specimen, whether samples were collected at diagnosis or follow-up and when post-diagnosis follow-up occurred.

**Fig 1:**
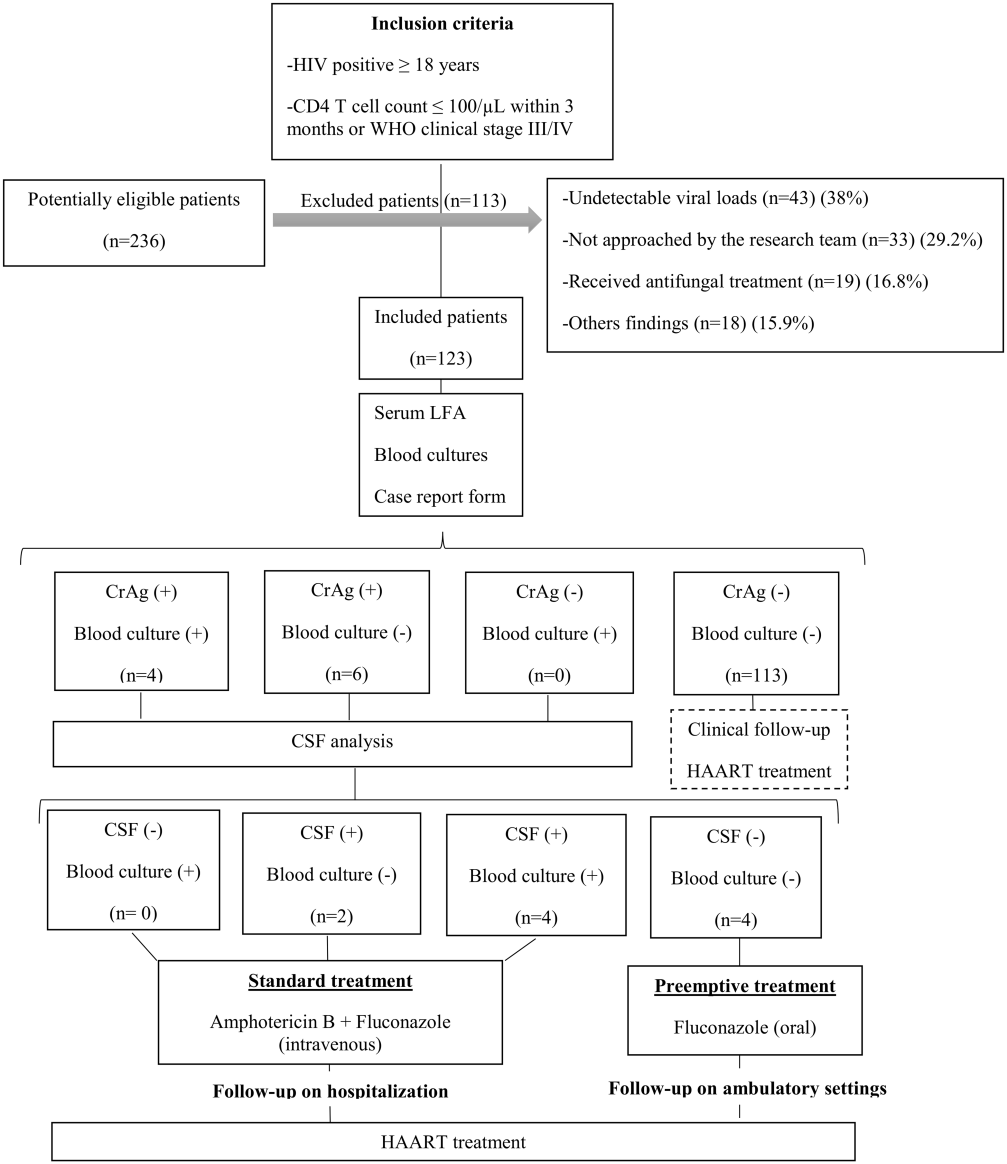
Flow chart of the specimen testing algorithm.

Subjects were tested as outlined in Fig 1. The blood samples were centrifuged for 10 minutes at 3,000 rpm and the LFA was performed according to the manufacturer’s instructions on all serum samples [10]. Additionally, 10 ml of blood was collected in a sterile syringe with heparin and 9 ml was aseptically transferred in a lysis centrifugation tube, containing 1 ml of 0.5% saponin. The tube was gently agitated to mix the contents with the inoculated blood, and was centrifuged to 3,000 rpm for 30 minutes. Pathogen identification of isolates from positive blood cultures was performed using standard microbiology methods (morphological and biochemical tests).

When serum CrAg detection was positive, a lumbar puncture was performed to rule out CM through India-ink testing on the CSF, CSF CrAg and culture [10]. Meningeal involvement was defined when one of the latter described methods was positive. Following current recommendations for countries where flucytosine is unavailable, patients with CM were treated with amphotericin B deoxycholate (0.7–1 mg/kg/day) and intravenous fluconazole (800 mg/day) with an induction phase of 14 days [7,8]. CrAg positive patients without evidence of meningeal involvement or disseminated cryptococcosis were treated for the same period of time with oral fluconazole 800 mg/day in ambulatory care. In both cases, the consolidation phase was completed with fluconazole 400–800 mg/day.

### Statistical analysis

Since this was an exploratory study, sample size was not determined in advance, and subject enrollment was set to include all eligible patients seen within a 10 month period, resulting in an estimated recruitment of 150 participants based on foreign and local studies [11–15]. Descriptive statistics were used to summarize the baseline characteristics of participants and prevalence rates. Categorical variables were described with n (%) and quantitative variables with mean and standard deviation (SD) or median and interquartile range (IQR) as appropriate. Parametric or non-parametric methods were used depending on the distribution of quantitative variables (Student's *t*-test or Mann-Whitney-Wilcoxon test). For categorical variables Chi squared or Fisher exact test was used, whichever was more appropriate. All statistical tests were considered significant if the alpha level was lower than 5%. Alpha levels below 10% were considered indicative of trends.

The number needed to screen was calculated to detect one patient with positive CrAg.

All data were anonymized and analyzed using the software EPI-Info 7.

## Results

Of the 236 patients who were likely potentially candidates for inclusion in this study, 113 (47.8%) were excluded for the reasons described in Fig 1.

We included 123 patients that were followed by a median time of 9 months (IQR 6–12 months) after the samples were taken. All of them completed the follow-up period set. Baseline characteristics of the patients tested are shown in Table 1. Prevalence of CrAg-positivity among included patients was 8.1% (CI95% 3.3–13.0) (Fig 1). As required by exclusion criteria, none of the CrAg-positive cases has history of CM. Among the 10 CrAg-positive patients, 6 had meningeal involvement detected through the CSF analysis though only 3 of them had neurologic symptoms at baseline. All received immediate standard treatment with amphotericin B deoxycholate and intravenous fluconazole. Two of these patients died during hospitalization. The remaining 4 patients with positive CrAg received targeted preemptive treatment with oral fluconazole and were free of cryptococcal disease and receiving HAART at the end of the follow-up period. None of the 113 patients with a negative CrAg test result developed cryptococcal disease.

**Table 1:** Baseline characteristics of the cohort^a^.

Variable	Total	CrAg (+)	CrAg (-)	P-value
N	123	10	113^b^	
**Gender**				0.403
Male	79 (64.2%)	5 (50%)	74 (65.5%)	
Female	38 (30.9%)	5 (50%)	33 (29.2%)	
Transwomen	6 (4.9%)		6 (5.3%)	
**Age, mean years (SD)**	38 (9.7)	38 (12.9)	38 (9.4)	0.958
**Time of HIV Diagnosis at Enrolment**				0.029
< 1 year	34 (27.8%)		34 (30.4%)	
1–5 years	20 (16.4%)	2 (20%)	18 (16%)	
5–10 years	27 (22.1%)	1 (10%)	26 (23.2%)	
>10 years	41 (33.6%)	7 (70%)	34 (30.4%)	
**Laboratory Finding**				
CD4 cell count, median cells/mL (IQR)	46 (18–85)	38 (14–105)	46 (19–84)	0.672
Viral load, median copies/mL (IQR)	65,609 (21,943–156,299)	126,468 (32,220–268,637)	65,609 (21,943–146,883)	0.371
**WHO stage III/IV**	98 (79.6%)	9 (90%)	91 (80.5%)	0.462
**Previous opportunistic infection**	60 (48.7%)	8 (80%)	52 (46%)	0.039
**Without HAART**	95 (77.2%)	9 (90%)	86 (76.1%)	0.315

**Abbreviations**: HAART, highly active antiretroviral therapy; CrAg, cryptococcal antigenemia; IQR, interquartile; SD, standard deviation; WHO, World Health Organization.

^a^ Data are presented as n (%) or median (IQR) or mean (SD).

^b^ Data was missing for time of HIV diagnosis at enrolment (n = 1).

The number needed to screen for detecting one positive patient for CrAg LFA was 12.

## Discussion

In this prospective study we report a prevalence rate 8.1% of CrAg among patients with advanced HIV infection. Routine screening provided the early detection of 10 cases, including 6 cases of CM, half of them without neurological symptoms.

Despite the advent of HAART, *Cryptococcus* remain a significant disease among HIV-infected persons, primarily in low and middle-income countries [16]. Data about the burden of cryptococcal disease in Latin America and Argentina are scarce. The overall prevalence of positive serum CrAg in the present study is similar to that reported by other authors in Uganda, with a prevalence rate of 8.8%, among patients with a CD4+ cell count <100 cells/mL [12]. These prevalence rates are lower than to those from Cambodia and South Africa among patients with similar demographic characteristics [17,18] and are three times greater than those reported from the United States, using the same assessment methods [19].

Routine screening might identify asymptomatic CM. Symptom-based diagnosis is not a reliable predictor of central nervous system involvement [20]. In our study through performing lumbar puncture in all patients with CrAg positive, we identified CM in 3 asymptomatic patients, supporting the recommendations of Vidal [1]. If we had performed CrAg only in those with symptoms such as fever or headache, we could have missed 50% of the diagnosis. In addition, the low number needed to screen to identify a positive case favors the routine use of CrAg among advanced patients. This number was similar to that reported in an observational study conducted in Uganda among HAART-naïve, HIV-infected patients with a CD4 T-cell count <100 cells/μL [12].

According to the results showed in the table, one quarter of individuals were recently diagnosed at such advanced stage. Late HIV diagnosis remains a problem in all Latin America [21].

Many studies have reported the diagnostic value of the LFA in detecting CrAg. A recently published paper demonstrates that LFA in serum and CSF is highly predictive of cryptococcosis among patients at risk for this diagnosis [22]. The presence of asymptomatic antigenemia is a recognized risk factor for the developing of immune reconstitution inflammatory syndrome [23] and the overwhelming risk factor for HAART-associated cryptococcosis, as patients with untreated antigenemia are more likely to develop clinical cryptococcosis [24].

In the HIV-positive patient, CM is a late opportunistic infection, usually observed when the T lymphocyte CD4+ cell count falls below 50–100/μL, leading to an acute mortality in the developing world of about 13% to 55% [4,9,25]. In patients with cryptococcal disease, CrAg is detectable a median of 22 days before symptoms onset [11]. This antigenemia is rare in patients with CD4 > 100/μL and the evidence from animal studies suggests that the presence of serum CrAg is a manifestation of extrapulmonary disease [18,26].

Although the screening strategy used in our study has not been formally evaluated in Latin America, epidemiological data suggest its potential benefit and cost-savings [1]. We confirmed the clinical utility of LFA for the rapid diagnosis of cryptococcosis and assessed its potential use as a point of care test. Although direct microscopic examination and culture, remain essential for the diagnosis of this infection, its sensitivity is limited. India-ink testing depends on the quality and quantity of the sample and intensive observer training. Cultures requires several days of incubation for development, making their use as early diagnostic techniques impractical [27]. The LFA is an ideal point of care test, as it can be performed by providers with minimal training, and without any additional laboratory equipment other than a tube to hold the specimen. Only 1 drop of bodily fluid is required, and the assay can be performed at room temperature, not requiring refrigeration or heat inactivation [28]. Another advantage of LFA is that it can be performed on remanent blood samples used for routine testing, reducing the need of additional visits. Prices varies according demand, competition and other market dynamics. While in Africa the price for the CrAg LFA is about USD 2.5 in our country the price is USD 6 per test. It is expectable that, if implemented widely, the price should drop, increasing cost-effectiveness analysis.

Early diagnosis of CM would result in a reduction in CM-related deaths as patients who receive early antifungal treatment have been shown to have better outcomes than those who receive delayed treatment [12]. CrAg positive patients without meningeal involvement and disseminated disease who received induction therapy with fluconazole preventive oral, not only prevent the progression of their disease, but also the hospitalization and administration of intravenous drugs, with a corresponding reduction in costs.

Only four patients were CrAg positive, without evidence of spread or meningeal involvement, who were free of disease with preemptive therapy in the follow-up period.

Different studies support the implementation of routinely detect and treat asymptomatic cryptococcal antigenemia among individuals initiating HAART, even with low doses of fluconazole treatment, showing a reduction of the mortality [12,29,30].

The high prevalence in our study supports the WHO guidelines [8] and call for a local recommendation for routine CrAg screening among HIV advanced patients followed by preemptive fluconazole therapy.

Our study had some limitations. About 30% of potentially eligible subjects were not approached. These missed screening opportunities may have resulted in an under estimation of the actual prevalence rate. Other potential limitation of our study include its relatively small sample size. However, this is the first study in Argentina, to our knowledge, describing the prevalence of cryptococcosis and usefulness of their screening.

We conclude that LFA provided rapid diagnosis to determine a high prevalence of CrAg in our hospital, and that screening for subclinical infection with preemptive antifungal treatment, prevented a substantial proportion of meningeal disease. The use of point-of-care tests like LFA played an important role in early diagnosis of cryptococcosis in advanced HIV patients, justifying its implementation. We expect that earlier diagnosis would result in lower fungal loads and a reduction in mortality. In countries like Argentina, where resources and access to HAART are universally available but HIV diagnoses are still delayed, the development of a rapid, sensitive and specific point-of-care test to detect cryptococcal infection will allow its early diagnosis and treatment.

## Supporting information

S1 File Database. (DOCX)
