# Supplementary material for: Prevalence of cryptococcal infection among advanced HIV patients in Argentina using lateral flow immunoassay
Source: PLoS One. 2017 Jun 15;12(6):e0178721. doi: 10.1371/journal.pone.0178721 (PMC5472272; doi:10.1371/journal.pone.0178721)
Supplement: Supplementary file 1 [file pone.0178721.s001.docx]

ID	Sexo	Genero	Edad	CD4	CV	Dx_HIV	TARV	Sint_Neuro_	Ag_suero_Cr	CrAg_Tit_suer	Ag_LCR-Cryp	CrAg_Tit_LCI	Hemo_Crypt	LCR_cult_Cry	Otras_Mx_C	Obito	X Crypto	Seguim (meses)
1	M	M	36	12	S/D	feb-14	NO	NO	negativo		no tiene		negativo	no tiene	NO	NO		13
2	M	M	60	21	S/D	jul-04	NO	NO	POSITIVO	1/100	POSITIVO	1/100	POSITIVO	POSITIVO	NO	NO		13
3	M	M	45	65	29798	mar-05	NO	NO	negativo		no tiene		negativo	no tiene	NO	NO		13
4	M	M	37	S/D	S/D	jul-96	NO	NO	negativo		no tiene		negativo	no tiene	NO	NO		13
5	M	M	31	44	6178	ago-08	SI	NO	negativo		no tiene		negativo	no tiene	NO	NO		13
6	M	M	50	30	81679	dic-12	NO	NO	negativo		no tiene		negativo	no tiene	NO	NO		13
7	F	F	44	86	106524	oct-13	NO	NO	negativo		no tiene		negativo	no tiene	NO	SI	NO	2
8	M	M	50	S/D	S/D	jul-12	NO	NO	negativo		no tiene		negativo	no tiene	NO	SI	NO	10
9	M	M	18	25	150959	ago-95	NO	NO	negativo		no tiene		negativo	no tiene	NO	NO		13
10	F	F	38	43	52717	mar-02	NO	NO	negativo		no tiene		negativo	no tiene	NO	NO		13
11	M	M	25	160	178436	nov-13	NO	NO	negativo		no tiene		negativo	no tiene	NO	NO		13
12	M	M	29	104	55230	mar-14	NO	NO	negativo		no tiene		negativo	no tiene	NO	NO		13
13	M	M	20	44	111000	ago-93	NO	NO	negativo		no tiene		negativo	no tiene	NO	NO		13
14	F	F	44	113	41954	oct-10	NO	NO	negativo		no tiene		negativo	no tiene	NO	NO		13
15	F	F	40	31	27862	jun-97	NO	NO	negativo		no tiene		negativo	no tiene	NO	NO		13
16	F	F	47	7	2634	jul-06	NO	NO	negativo		no tiene		negativo	no tiene	NO	NO		13
17	F	F	29	56	103621	ene-07	NO	NO	negativo		no tiene		negativo	no tiene	NO	NO		13
18	M	M	27	46	S/D	jul-13	NO	NO	negativo		no tiene		negativo	no tiene	NO	NO		13
19	M	M	45	70	14431	jul-92	NO	SI	POSITIVO	1/10	POSITIVO	1/10	POSITIVO	POSITIVO	NO	NO		13
20	F	F	30	0	1779	ene-14	SI	NO	negativo		no tiene		negativo	no tiene	NO	SI	NO	7
21	M	M	41	370	3924	mar-14	NO	NO	negativo		no tiene		negativo	no tiene	NO	NO		12
22	M	M	33	46	138942	jul-98	NO	NO	negativo		no tiene		negativo	no tiene	NO	NO		12
23	F	F	43	74	110000	jun-09	NO	NO	negativo		no tiene		negativo	no tiene	NO	NO		12
24	M	M	49	104	S/D	jun-99	NO	NO	negativo		no tiene		negativo	no tiene	NO	NO		12
25	F	F	32	132	34375	jul-06	NO	NO	negativo		no tiene		negativo	no tiene	NO	NO		12
26	F	F	35	27	29574	jul-01	NO	NO	negativo		no tiene		negativo	no tiene	NO	SI	NO	<1
27	M	M	36	242	117996	jul-94	NO	NO	negativo		no tiene		negativo	no tiene	NO	NO		12
28	M	M	56	17	S/D	may-14	NO	NO	negativo		no tiene		negativo	no tiene	NO	NO		12
29	M	M	38	29	38538	oct-08	SI	NO	negativo		no tiene		negativo	no tiene	NO	SI	NO	2
30	M	M	49	14	S/D	abr-14	NO	NO	negativo		no tiene		negativo	no tiene	NO	SI	NO	12
31	M	M	56	108	8057	jul-91	NO	NO	negativo		no tiene		negativo	no tiene	NO	NO		12
32	F	F	20	3	279287	oct-11	SI	NO	POSITIVO	1/10	NEGATIVO		negativo	NEGATIVO	NO	NO		12
33	F	F	42	98	S/D	feb-14	SI	NO	negativo		no tiene		negativo	no tiene	NO	NO		12
34	F	F	33	5	121002	ago-04	SI	NO	negativo		no tiene		negativo	no tiene	NO	NO		12
35	M	M	35	17	349238	sep-06	SI	NO	negativo		no tiene		negativo	no tiene	NO	NO		12
36	M	M	34	19	83800	may-06	NO	NO	negativo		no tiene		negativo	no tiene	NO	NO		12
37	M	M	32	S/D	23378	mar-02	NO	NO	negativo		no tiene		negativo	no tiene	NO	NO		12
38	M	M	36	10	75586	jun-00	NO	NO	negativo		no tiene		negativo	no tiene	NO	NO		12
39	F	F	49	1	S/D	jul-93	NO	NO	negativo		no tiene		negativo	no tiene	NO	NO		12
[illegible]	[illegible]	[illegible]	[illegible]	[illegible]	[illegible]	[illegible]	[illegible]	[illegible]	[illegible]		[illegible]		[illegible]	[illegible]	[illegible]	[illegible]		[illegible]

41	F	F	42	170	500000	jul-96	NO	SI	POSITIVO	1/100	POSITIVO	1/10	POSITIVO	POSITIVO	NO	NO		12
42	M	F	37	29	S/D	jul-06	NO	NO	negativo		no tiene		negativo	no tiene	NO	NO		12
43	M	M	49	4	277	jul-97	NO	NO	negativo		no tiene		negativo	no tiene	NO	NO		12
44	M	M	34	73	75848	mar-12	NO	NO	negativo		no tiene		negativo	no tiene	NO	NO		12
45	F	F	34	105	205640	feb-08	NO	NO	POSITIVO	1/10	NEGATIVO		negativo	POSITIVO	NO	SI	SI	8
46	M	M	43	264	40054	jul-92	NO	NO	negativo		no tiene		negativo	no tiene	NO	NO		12
47	F	F	22	S/D	S/D	dic-08	NO	NO	negativo		no tiene		negativo	no tiene	NO	NO		11
48	M	M	51	24	35415	jul-98	NO	NO	negativo		no tiene		negativo	no tiene	NO	NO		11
49	F	F	38	4	S/D	jul-01	NO	NO	negativo		no tiene		negativo	no tiene	NO	SI	NO	3
50	M	M	39	78	21943	jul-91	SI	NO	negativo		no tiene		negativo	no tiene	NO	NO		11
51	F	F	43	14	17341	dic-03	NO	NO	POSITIVO	1/10	NEGATIVO		negativo	NEGATIVO	NO	NO		11
52	M	M	31	28	26132	jun-09	NO	NO	negativo		no tiene		negativo	no tiene	NO	NO		11
53	M	M	29	145	72233	jul-05	SI	NO	negativo		no tiene		negativo	no tiene	NO	NO		11
54	F	F	37	15	485076	jul-99	SI	NO	negativo		no tiene		negativo	no tiene	NO	NO		11
55	M	F	46	108	S/D	jul-12	SI	NO	negativo		no tiene		negativo	no tiene	NO	SI	NO	<1
56	F	F	33	257	52934	dic-11	NO	NO	negativo		no tiene		negativo	no tiene	NO	NO		11
57	F	F	40	47	38376	jun-97	SI	NO	negativo		no tiene		negativo	no tiene	NO	SI	NO	5
58	M	M	34	S/D	S/D	jun-14	NO	NO	negativo		no tiene		negativo	no tiene	NO	NO		11
59	F	F	42	22	353651	may-94	NO	NO	negativo		no tiene		negativo	no tiene	NO	NO		10
60	F	F	45	S/D	S/D	jun-14	NO	NO	negativo		no tiene		negativo	no tiene	NO	NO		10
61	M	M	35	78	123	nov-13	NO	NO	negativo		no tiene		negativo	no tiene	NO	NO		10
62	M	M	32	19	S/D	jul-14	NO	NO	negativo		no tiene		negativo	no tiene	NO	NO		10
63	F	F	24	26	263317	jul-08	SI	NO	negativo		no tiene		negativo	no tiene	NO	NO		10
64	M	M	36	64	8791	jul-94	SI	NO	negativo		no tiene		negativo	no tiene	NO	NO		10
65	M	M	38	85	204573	jun-14	NO	NO	negativo		no tiene		negativo	no tiene	NO	NO		10
66	M	M	31	6	22185	nov-04	SI	NO	negativo		no tiene		negativo	no tiene	NO	SI	NO	1
67	M	M	48	S/D	S/D	jul-14	NO	NO	negativo		no tiene		negativo	no tiene	NO	SI	NO	2
68	F	F	41	41	477541	jul-14	NO	NO	negativo		no tiene		negativo	no tiene	NO	NO		9
69	M	M	32	84	154708	jul-09	NO	NO	negativo		no tiene		negativo	no tiene	NO	NO		9
70	M	M	50	75	172413	jul-88	NO	NO	negativo		no tiene		negativo	no tiene	NO	NO		9
71	M	M	51	149	11021	may-87	NO	NO	negativo		no tiene		negativo	no tiene	NO	NO		9
72	M	F	27	214	4900	ago-06	NO	NO	negativo		no tiene		negativo	no tiene	NO	NO		9
73	M	F	33	67	3679	ene-14	NO	NO	negativo		no tiene		negativo	no tiene	NO	NO		9
74	M	M	36	10	288618	jul-03	NO	NO	negativo		no tiene		negativo	no tiene	NO	NO		9
75	M	M	56	61	S/D	jun-02	NO	NO	negativo		no tiene		negativo	no tiene	NO	NO		9
76	M	M	30	55	47297	mar-03	NO	NO	POSITIVO	Título puro	NEGATIVO		negativo	NEGATIVO	NO	NO		9
77	M	M	60	10	157891	may-14	NO	NO	negativo		no tiene		negativo	no tiene	NO	NO		8
78	M	M	42	66	500000	ago-14	NO	NO	negativo		no tiene		negativo	no tiene	NO	NO		8
79	M	M	43	43	11492	ene-04	NO	NO	negativo		no tiene		negativo	no tiene	NO	NO		8
80	F	F	25	35	32146	jul-91	NO	NO	negativo		no tiene		negativo	no tiene	NO	NO		8

81	M	M	49	2	25693	nov-08	SI	NO	negativo		no tiene		negativo	no tiene	NO	NO		8
82	F	F	38	20	10275	jul-11	NO	NO	negativo		no tiene		negativo	no tiene	NO	NO		8
83	M	M	41	17	S/D	jul-14	NO	NO	negativo		no tiene		negativo	no tiene	NO	NO		8
84	F	F	52	78	8523	jul-07	NO	NO	negativo		no tiene		negativo	no tiene	NO	NO		8
85	M	M	32	80	S/D	abr-06	SI	NO	negativo		no tiene		negativo	no tiene	NO	NO		8
86	M	M	34	86	500000	sep-14	NO	NO	negativo		no tiene		negativo	no tiene	NO	NO		7
87	M	M	29	226	S/D	jul-12	NO	NO	negativo		no tiene		negativo	no tiene	NO	NO		7
88	F	F	23	35	10101	jun-14	SI	NO	negativo		no tiene		negativo	no tiene	NO	NO		7
89	F	F	40	8	137211	ene-09	SI	NO	negativo		no tiene		negativo	no tiene	NO	NO		7
90	M	F	26	45	146883	oct-14	NO	NO	negativo		no tiene		negativo	no tiene	NO	NO		7
91	M	M	29	4	500000	S/D	NO	NO	negativo		no tiene		negativo	no tiene	NO	NO		7
92	F	F	21	7	47100	mar-93	NO	NO	POSITIVO	1/100	POSITIVO	1/1000	POSITIVO	POSITIVO	ESCARIFICAC	SI	SI	<1
93	M	M	52	16	S/D	ago-99	NO	SI	POSITIVO	1/100	POSITIVO	1/1000	negativo	POSITIVO	NO	NO		7
94	M	M	45	61	95200	ene-06	NO	NO	negativo		no tiene		negativo	no tiene	NO	NO		7
95	F	F	39	5	122332	oct-14	NO	NO	negativo		no tiene		negativo	no tiene	NO	NO		7
96	M	M	43	65	S/D	sep-14	NO	NO	negativo		no tiene		negativo	no tiene	NO	NO		7
97	M	M	39	S/D	S/D	jul-94	SI	NO	negativo		no tiene		negativo	no tiene	NO	NO		7
98	M	M	36	8	S/D	oct-14	NO	NO	negativo		no tiene		negativo	no tiene	NO	NO		6
99	M	M	44	81	145000	ene-05	NO	NO	negativo		no tiene		negativo	no tiene	NO	NO		6
100	M	F	35	16	58986	nov-13	NO	NO	negativo		no tiene		negativo	no tiene	NO	NO		6
101	M	M	43	5	S/D	jul-01	NO	NO	negativo		no tiene		negativo	no tiene	NO	NO		6
102	M	M	40	S/D	S/D	jul-05	NO	NO	negativo		no tiene		negativo	no tiene	NO	NO		6
103	M	M	32	106	257988	ago-11	NO	NO	POSITIVO	Título puro	NEGATIVO		negativo	NEGATIVO	NO	NO		6
104	M	M	27	26	S/D	sep-14	SI	NO	negativo		no tiene		negativo	no tiene	NO	NO		6
105	M	M	43	67	500000	nov-14	NO	NO	negativo		no tiene		negativo	no tiene	NO	NO		6
106	M	M	35	S/D	S/D	jul-10	NO	NO	negativo		no tiene		negativo	no tiene	NO	NO		5
107	M	M	28	49	500000	oct-14	NO	NO	negativo		no tiene		negativo	no tiene	NO	NO		5
108	M	M	66	33	S/D	nov-14	NO	NO	negativo		no tiene		negativo	no tiene	NO	NO		5
109	M	M	26	S/D	S/D	dic-14	NO	NO	negativo		no tiene		negativo	no tiene	NO	NO		5
110	M	M	36	S/D	S/D	jul-13	NO	NO	negativo		no tiene		negativo	no tiene	NO	NO		5
111	M	M	39	S/D	S/D	jun-12	NO	NO	negativo		no tiene		negativo	no tiene	NO	NO		5
112	F	F	24	258	S/D	may-90	SI	NO	negativo		no tiene		negativo	no tiene	NO	NO		5
113	F	F	66	281	2028	abr-04	SI	NO	negativo		no tiene		negativo	no tiene	NO	NO		5
114	F	F	39	46	72528	jun-06	SI	NO	negativo		no tiene		negativo	no tiene	NO	NO		5
115	M	M		S/D	S/D	ene-15	NO	NO	negativo		no tiene		negativo	no tiene	NO	NO		4
116	M	M	20	270	S/D	dic-14	NO	NO	negativo		no tiene		negativo	no tiene	NO	NO		4
117	F	F	42	77	242	jul-05	SI	NO	negativo		no tiene		negativo	no tiene	NO	NO		4
118	F	F	27	141	4900	feb-08	SI	NO	negativo		no tiene		negativo	no tiene	NO	NO		4
119	M	M	31	S/D	S/D	ene-15	NO	NO	negativo		no tiene		negativo	no tiene	NO	NO		4
120	M	M	24	30	S/D	dic-14	SI	NO	negativo		no tiene		negativo	no tiene	NO	SI	NO	<1
121	M	M	28	S/D	S/D	ene-15	NO	NO	negativo		no tiene		negativo	no tiene	NO	NO		4
